# Molecular Determinants of Sensitivity or Resistance of Cancer Cells Toward Sanguinarine

**DOI:** 10.3389/fphar.2018.00136

**Published:** 2018-02-26

**Authors:** Mohamed E. M. Saeed, Nuha Mahmoud, Yoshikazu Sugimoto, Thomas Efferth, Heba Abdel-Aziz

**Affiliations:** ^1^Department of Pharmaceutical Biology, Johannes Gutenberg University, Mainz, Germany; ^2^Division of Chemotherapy, Faculty of Pharmacy, Keio University, Tokyo, Japan; ^3^Medical and Clinical Affairs Phytomedicines, Steigerwald Arzneimittelwerk GmbH, Bayer Consumer Health, Darmstadt, Germany

**Keywords:** bioinformatics, cancer, drug resistance, microarray, pharmacogenomics, phytotherapy

## Abstract

For decades, natural products represented a significant source of diverse and unique bioactive lead compounds in drug discovery field. In Clinical oncology, complete tumors remission is hampered by the development of drug-resistance. Therefore, development of cytotoxic agents that may overcome drug resistance is urgently needed. Here, the natural benzophenanthridine alkaloid sanguinarine has been studied for its cytotoxic activity against multidrug resistance (MDR) cancer cells. We investigated the role of the ATP-binding cassette (ABC) transporters BCRP/ABCG2, P-glycoprotein/ABCB1 and its close relative ABCB5 in drug resistance. Further drug resistance mechanisms analyzed in this study were the tumor suppressor TP53 and the epidermal growth factor receptor (EGFR). Multidrug resistant cells overexpressing BCRP, ABCB5 and mutated ΔEGFR were not cross-resistant toward sanguinarine. Interestingly, P-gp overexpressing cells were hypersensitive to sanguinarine. Doxorubicin uptake assay carried by flow cytometry revealed that sanguinarine is a potent inhibitor of the P-gp transporter. Moreover, immunoblotting analysis proved that P-gp was downregulated in a dose dependent manner after treating P-gp overexpressing cells with sanguinarine. It was surmised that The inhibition of NFκB activity might explain the collateral sensitivity in CEM/ADR5000 cells. The COMPARE and hierarchical cluster analyses of transcriptome-wide expression profiles of tumor cell lines of the National Cancer Institute identified genes involved in various cellular processes (immune response, inflammation signaling, cell migration and microtubule formation) significantly correlated with log_10_IC_50_ values for sanguinarine. In conclusion, sanguinarine may have therapeutic potential for treating multidrug resistant tumors.

## Introduction

Sanguinarine is a natural benzophenanthridine alkaloid isolated from *Sanguinaria canadensis* (Papaveraceae). The roots of the plant, also known as bloodroot because of its red latex, are widely used in North America for cold, cough, sore throat, as a systemic expectorant, antifungal, and ulcers of the skin (Predny and Chamberlain, 2005; Croaker et al., 2016). The plant is applied topically in paste form to treat cancerous growths, warts, boils and polyps in the skin (Howell, 2006).

Sanguinarine exerts several biological activities including, antimicrobial, antioxidant, anti-inflammatory as well as anticancer properties (Jeng et al., 2007). The cytotoxicity of sanguinarine against tumor cells has been reported *in vitro* and *in vivo* (Gaziano et al., 2016; Bodle et al., 2017; Wei et al., 2017). Furthermore, sanguinarine induced apoptosis and triggered cell death signaling cascades in numerous cancer cell lines (Lee et al., 2008, 2016; Xu et al., 2012). Several modes of action have been proposed to explain the anticancer activity of sanguinarine, such as activation of the caspase machinery in mitochondria, irreversible microtubule depolymerization, depletion of nuclear topoisomerase II preventing DNA strand break reconnection, inhibition of B-DNA to Z-DNA transition altering DNA supercoiling, capping telomeres inducing rapid apoptosis, depletion of cellular glutathione, and cell cycle arrest (Rich, 1994; Das et al., 1999; Messori et al., 2001; Debiton et al., 2003; Adhami et al., 2004; Bai et al., 2006, 2008; Lopus and Panda, 2006; Nitiss, 2009). Moreover, sanguinarine targets vital cellular compartments including extracellular signal-regulated kinases and NF-κB, which are involved in signal transduction pathways associated with cell proliferation and/or cell death mechanisms (Lee et al., 2008). In addition, it has been shown that sanguinarine inhibits the sodium–potassium ATPase affecting the membrane permeability (Ding et al., 2002).

Drug resistance to clinically established chemotherapeutic agents remains the main obstacle toward achieving complete tumors remission and long-lasting cures. Several mechanisms have been proposed to be responsible for chemotherapy failure. Multidrug resistance (MDR) is the phenomenon in which cancer cells exhibit cross-resistance or loss the sensitivity to various chemotherapeutic agents that are structurally and functionally different, including anthracyclines, Vinca alkaloids, colchicine, epipodophyllotoxins, taxanes, camptothecins, imatinib, and mitoxantrone (Raaijmakers et al., 2006; Efferth et al., 2008; Fletcher et al., 2010). Well-known mechanisms with clinical significance represent the overexpression of transmembrane efflux pumps such as ATP-binding cassette (ABC) transporters (P-gp, BCRP and ABCB5), activation of enzymes of the glutathione detoxification system, mutations in genes involved in tumor development and apoptosis (e.g., EGFR and p53). These mechanisms act in a cooperative manner due to tumor heterogeneity. Therefore, MDR is defined as a multifactorial process. Despite most mechanisms of MDR have been well studied, understanding their role in the clinical setting still represents a significant challenge.

Chemotherapy is considered as a corner stone in cancer treatment worldwide. The development of MDR and the severe side effects are common challenges for the therapeutic efficacy of most clinically used chemotherapeutic agents. Therefore, the identification of novel effective therapeutic agents is urgently needed. In this context, phytochemicals may represent an attractive alternative because of their affordability and low toxicity (Efferth, 2017; Efferth et al., 2017).

The objectives of the present study were, firstly, to investigate whether ABC transporters (P-gp, ABCB5, BCRP) as classical MDR mechanisms also play a role in response to sanguinarine. Secondly, we addressed the question whether the cytotoxic activity of sanguinarine correlated to other molecular determinants in the cell line panel of the National Cancer Institute (NCI, USA). In addition to ABC transporters, we also studied oncogenes and tumor suppressor genes (EGFR, TP53). Furthermore, we performed bioinformatic COMPARE and hierarchical cluster analyses of microarray-based transcriptomic mRNA expression data of the NCI cell lines that were correlated to sanguinarine's response, as well as transcription factor promoter binding motif analysis searching for common transcription factors that might regulate the genes appeared in microarray-and COMPARE analyses. Moreover, we carried *in vitro* assay detecting the activity of drug resistance-mediating transcription factor NFκB using cells stably expressing reporter gene preceded by NFκB promoter, as well as *in silico* molecular docking analyses of sanguinarine to NFκB and its regulator IκB.

## Materials and methods

### Cell lines

Two leukemia cell lines, drug-sensitive CCRF-CEM and multidrug-resistant CEM/ADR5000, were cultured in RPMI 1640 medium, supplemented with 10% fetal bovine serum (FBS) (Invitrogen, Darmstadt, Germany) and 1% penicillin (100 U/ml)-streptomycin (100 μg/ml) antibiotic (Invitrogen, Darmstadt, German) and incubated in humidified 5% CO_2_ atmosphere at 37°C. Human HEK293-ABCB5 embryonic kidney cells transfected with another ABC-transporter, ABCB5, were propagated in DMEM medium supplemented with 10% FBS and 1% penicillin/streptomycin (Invitrogen) (Kawanobe et al., 2012). Breast cancer cells transduced with control vector (MDA-MB-231-pcDNA3) or with a cDNA for the breast cancer resistance protein BCRP (MDA-MB-231BCRP clone 23), human wild-type HCT116 (p53^+/+^) colon cancer cells as well as knockout clones HCT116 (p53^−/−^) derived by homologous recombination, non-transduced human U87MG glioblastoma multiforme cells and U87MG cells transduced with an expression vector harboring an epidermal growth factor receptor (EGFR) gene with a genomic deletion of exons 2 through 7 (U87MG.ΔEGFR) were all maintained in DMEM medium, supplemented with 10% FBS and 1% penicillin-streptomycin and incubated under standard conditions as described for leukemia cell lines. The resistance of the different resistant cell lines has been maintained by adding 5,000 ng/ml doxorubicin for CEM/ADR5000, 400 μg/ ml geneticin for U87MG.ΔEGFR and HCT116 (p53^−/−^) and 800 ng/ml of the same compound for MDA-MB-231 BCRP clone 23.

A panel of 59 human tumor cell lines of the Developmental Therapeutics Program of the NCI (Bethesda, MD, USA) consisted of leukemia, melanoma, non-small cell lung cancer, colon cancer, renal cancer, ovarian cancer, breast cancer, and prostate carcinoma cells as well as tumor cells of the central nervous system (Alley et al., 1988). Cells treated with sanguinarine for 48 h and cytotoxicity was evaluated using a sulforhodamine B assay (Rubinstein et al., 1990).

### Resazurin assay

The non-fluorescent dye resazurin is reduced metabolically by living cells to the strongly-fluorescent dye resorufin (O'Brien et al., 2000). The resazurin (Promega, Mannheim, Germany) reduction assay was performed to assess the cytotoxicity of sanguinarine toward drug-sensitive and -resistant cell lines. Briefly, tumor cells (2 × 10^4^cells/well) were seeded in 96-wells plate in a volume of 100 μL, and varying concentrations of sanguinarine were added to reach the total volume of 200 μL. After 72 h, 20 μL of 0.01% w/v resazurin (Sigma-Aldrich, Schnelldorf, Germany) was added to each well. Cells were incubated for 4 h at 37°C. Fluorescence at excitation wave length 544 nm and emission at 590 nm was measured using Infinite M2000 Pro™ plate reader (Tecan, Crailsheim, Germany). The percentage of viable cells was calculated as follows:

$$\begin{array}{l} \text{Cell Viability }\left(\text{\% of control}\right)=\\ \frac{\;absorption\;\left(treated\;cells\right)\;-\;absorption\;\left(medium\;alone\right)}{absorption\;\left(untreated\;cells\right)\;-\;absorption\;\left(medium\;alone\right)\;}\;*\;100 \end{array}$$

IC_50_ values were calculated from concentration dependent curves using nonlinear regression analysis tool built in Prism 7 GraphPad software. All IC_50_ values are expressed as the mean ± standard deviation (SD). Each assay was repeated thrice independently with six replicates each.

### Toxicity of sanguinarine in normal cells

Using Histopaque® (Sigma-Aldrich, St. Louis, MO, USA), the human peripheral mononuclear cells (PMNC) were isolated from fresh blood samples of a healthy donor. In brief, three mL blood was layered with three mL Histopaque® and centrifuged (400 × g) for 30 min at 4°C. The buffy coat interface, containing lymphocytes and other mononuclear cells, was transferred into a new tube and washed several times. Isolated PMNCs were kept in Panserin 413 medium (PAN-Biotech, Aidenbach, Germany) supplemented with 2% phytohemagglutinin M (PHA-M, Life Technologies, Darmstadt, Germany). Afterwards, the resazurin assay was carried out as described above.

### Doxorubicin uptake assay

The uptake of doxorubicin with and without the addition of sanguinarine has been measured using BD FACSCalibur™ (Beckton Dickinson, GmbH, Heidelberg, Germany). Sensitive parental CCRF-CEM leukemia cells, as well as their resistance P-glycoprotein-expressing subline, CEM/ADR5000 were tested. 10^6^ cells/well were seeded in 12-well tissue culture plates (Becton-Dickinson, Heidelberg, Germany) in the incubation medium (RPMI 1640, Invitrogen™) without phenol red. Doxorubicin at a concentration of 20 μM (University Hospital Pharmacy, Mainz, Germany) was used in all sample wells. Verapamil (20 μM, Sigma Aldrich, Taufkirchen, Germany) was used as a known inhibitor of P-gp. Sanguinarine (1 μM, Sigma Aldrich) was used as the test compound. After incubation for at 37°C 24 h, the cells were centrifuged to discard the old incubation medium and resuspended in fresh medium. Mean fluorescence intensity (MFI) of doxorubicin was measured by blue laser at an excitation wave length of 488 nm, and the emitted light was collected with a band pass filter at 530/30 nm. Every 30,000 cells were counted per sample. Dead cells were eliminated by gating the living cells in the forward vs. the side scatter.

### COMPARE and hierarchical cluster analyses of microarray data

COMPARE analysis is a web-based algorithm correlating between transcriptome-wide mRNA expressions and drug response of the NCI cell line panel (https://dtp.cancer.gov). The method is based on Pearson's rank correlation coefficient (Paull et al., 1989). We performed COMPARE analysis between the IC_50_ values for sanguinarine and the microarray-based transcriptome-wide mRNA expression levels in the NCI cell lines. Standard and reverse COMPARE were conducted to determine the genes, which were associated with resistance or sensitivity to sanguinarine, respectively.

Using the CIMMINER program (https://discover.nci.nih.gov/cimminer/), We performed agglomerative hierarchical cluster analysis (WARD method) to cluster the mRNA expression of genes identified by COMPARE analysis, and the heatmap was prepared accordingly.

Pearson's correlation test was used to calculate significance values and rank correlation coefficients as a relative measure for the linear dependence of two variables. The chi square (χ^2^) test was performed using the Excel program to proof bivariate frequency distributions for pairs of nominal scaled variables for dependencies obtained from cluster analysis/heat mapping.

### Transcription factor gene promoter binding motif analysis

The top 40 genes that either directly or inversely correlated with log_10_IC_50_ values of the NCI cell lines in COMPARE analysis were subjected to binding motif analysis. Promoter sequences 25 kb long upstream of exon 1 of the corresponding genes were retrieved from UCSC Genome Browser Gene Sorter (http://genome.ucsc.edu). Browser Extensible Data (BED) files were created to screen for the possible binding motifs for each gene. Promoter sequences were screened using the SeqPos tool implemented in the Galaxy Cistrome software [72].

### Molecular docking

Molecular docking is a predictive algorithm to evaluate the mode of binding of ligands with target macromolecules. X-ray crystallography-based structures of IκB and NF-κB were obtained from Protein Data Bank (http://www.rcsb.org/pdb); IκBα (PDB ID: 1IKN), and NF-κB (PDB ID: 1NFI). To determine the binding geometry of sanguinarine to both targets, a grid box was then constructed to define docking spaces for each protein. Docking parameters were set to 250 runs and 2,500,000 energy evaluations for each cycle. Docking was performed three times independently by Autodock4 and with AutodockTools-1.5.7rc1 using the Lamarckian algorithm. The corresponding lowest binding energies and predicted inhibition constants were obtained from the docking log files (dlg). Mean ± SD of binding energies were calculated from three independent dockings. Visual Molecular Dynamics (VMD) was used to depict the docking poses of sanguinarine for each target protein.

### Reporter cell line

HEK-Blue-Null1 cells (Invivogen, San Diego, CA, USA) stably express an optimized secreted embryonic alkaline phosphatase (SEAP) reporter gene under the control of the IFN-β promoter fused to NFκB binding site. The protocol was previously described by us (Seo et al., 2016). NFκB activation was detected by measuring SEAP spectrophotometrically at 630 nm (Tecan Teader, Tecan Group Ltd., Maennedorf, Switzerland).

### Proteins analyses by SDS-PAGE and immunoblotting

CEM/ADR5000 leukemia cells (10^6^ cells/well) were treated with varying concentrations of sanguinarine, harvested after 24 h, and washed with PBS. Total proteins extraction protocol was previously described (Saeed et al., 2015). Protein concentrations were measured by NanoDrop1000 (PEQLAB, Erlangen, Germany). Twenty micrograms of protein were electrophoresed on 10% SDS-polyacrylamide gels. Using wet sandwich blotting, the proteins were transferred onto Polyvinylidene difluoride (Ruti®-PVDF) membrane (Millipore Corporation, Billerica, MA). Membranes were washed using Tris-buffered saline containing 0.5% Tween-20 (TBST), then blocked with 5% (w/v) bovine serum albumin in TBST for 1 h at room temperature. Membranes were incubated at 4°C overnight with primary antibodies including: P-gp (1:1000) (Thermoscientific, Darmstadt, Germany), NFκB (1:1000) (Cell Signaling Technology, Frankfurt, Germany), IκBα (1:1000) (Cell Signaling Technology, Frankfurt, Germany), and β-actin (1:2000) (Cell Signaling Technology, Frankfurt, Germany). After washing membranes three times with TBST, the blots were probed with horseradish peroxidase-linked IgG secondary antibodies (1:2000) for 2 h at room temperature. Finally, LuminataTM Classico Western HRP substrate (Merck Millipore, Schwalbach, Germany) was added for 5 min in the dark. Alpha Innotech FluorChem Q system (Biozym, Oldendorf, Germany) was used for documentation and band analysis (Zhao et al., 2015).

## Results

### Role of classical drug resistance mechanisms for sanguinarine

**Figure 1:**
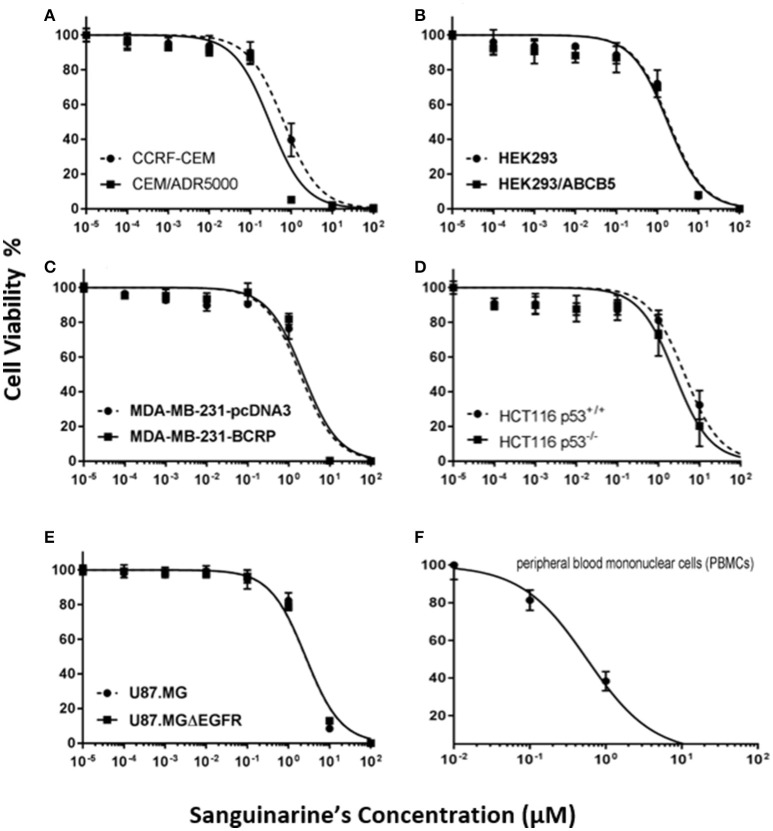
Dose response curves of sanguinarine. **(A)** Cytotoxicity of sanguinarine toward drug-sensitive parental CCRF-CEM tumor cells and their P-glycoprotein (MDR1/ABCB1)-expressing, multidrug-resistant subline, CEM/ADR5000. **(B)** Cytotoxicity of sanguinarine toward HEK293 cells and their ABCB5-transfectant subline, HEK293/ABCB5 as determined by resazurin assays. **(C)** Cytotoxicity of sanguinarine toward MDA-MB-231-pc DNA cells and their BCRP-transduced subline, MDA-MB-231-BCRP as determined by resazurin assays. **(D)** Cytotoxicity of sanguinarine toward HCT116 p53^+/+^ cells and their p53^−/−^ knockout subline, HCT116 p53^−/−^ as determined by resazurin assays. **(E)** Cytotoxicity of sanguinarine toward U87MG cells and their EGFR-transduced subline U87MGΔEGFR, as determined by resazurin assays. **(F)** Cytotoxicity of sanguinarine toward human peripheral mononuclear cells, as determined by resazurin assays.

By using the Pearson correlation test, we correlated the log_10_IC_50_ values of the NCI cell lines panel for sanguinarine with different of *mRNA* expression patterns of ABC transporters (*ABCB1, ABCG2*, and *ABCB5*), and *EGFR*, as well as mutations in the *TP53* or *H-, K-, N-RAS* genes - all of which represent known drug resistance mechanisms (Table 1). For each resistance mechanism, positive control drugs have been used. Interestingly, none of the expression patterns or mutations correlated with cellular response to sanguinarine. Indeed, these are favorable results which gave a clue that none of these classical drug resistance mechanisms may hamper the effect of sanguinarine in cancer cells. To confirm the results obtained by using the NCI cell line panel, we performed cytotoxicity assays of sanguinarine in sensitive and drug-resistant cell lines bearing the different drug resistance mechanisms. As shown in Figure 1 and Table 2, sanguinarine has been tested in concentrations range from 10^−5^ to 10^2^ μM. Interestingly, sanguinarine showed selective toxicity toward P-gp overexpressing cell lines and p53 knockout cell lines, when compared to their corresponding sensitive parent cell lines (resistance ratios of 0.5 and 0.65, respectively) (Figures 1A,D), a phenomenon called collateral sensitivity. Cross-resistance was not observed in the cell lines overexpressing other ABC transporters (ABCB5 and ABCG2) or mutation-activated EGFR, if treated with sanguinarine (Figures 1B,C,E). The sanguinarine inhibited the human peripheral mononuclear cells in 0.611 ± 0.1 μM. This represents two times concentration needed to kill resistant leukemia cells (CEM/ADR5000) (Figure 1F), which considered comparable counterpart as a tumor model.

**Table 1:** Correlation of log_10_IC_50_ values for sanguinarine to drug resistance mechanisms (ABCB1, ABCB5, ABCC1, ABCG2, EGFR, TP53, pan-RAS) in the NCI cell line panel.

**Resistance mechanism**	**Cell line**	**IC50 (μM)**	**Resistance ratio**
ABCB1	CCRF-CEM	0.6 ± 0.3	0.50
	CEM/ADR5000	0.3 ± 0.1	
EGFR	U87MG	2.6 ± 0.3	0.96
	U87ΔEGFR	2.5 ± 0.1	
BCRP	MDA231	2.1 ± 0.3	1.10
	MDA-BCRP	2.3 ± 0.1	
p53	p53^+/+^	4.1 ± 1.5	0.66
	p53^−/−^	2.7 ± 1.5	
ABCB5	HEK293	2.2 ± 0.4	1.00
	HEK293/ABCB5	2.2 ± 0.1	

*IC_50_ values are represented by mean ± SD of three independent experiments with each 6 parallel measurements*.

**Table 2:** IC_50_ values of different drug-sensitive and -resistant cell lines of Sanguinarine.

		**Sanguinarine (log_10_ IC_50_, M)**	**Control drug (log_10_ IC_50_, M)**
**ABCB1 Expression:**			**Daunorubicin**
7q21 (Chromosomal	*R*-value	0.063	^*^0.597
Locus of ABCB1 Gene)	*P*-value	0.327	^*^4.82 × 10^−6^
ABCB1 Expression	*R*-value	0.082	^*^0.684
(Microarray)	*P*-value	0.271	^*^1.57 × 10^−8^
ABCB1 Expression	*R*-value	0.076	^*^0.579
(RT-PCR)	*P*-value	0.297	^*^4.19 × 10^−6^
Rhodamine 123	*R*-value	0.010	^*^0.544
Accumulation	*P*-value	0.471	^*^1.51 × 10^−5^
**ABCB5 Expression:**			**Maytansine**
ABCB5 Expression	*R*-value	0.069	^*^0.454
(Microarray)	*P*-value	0.299	^*^6.67 × 10^−4^
ABCB5 Expression	*R*-value	−0.021	^*^0.402
(RT-PCR)	*P*-value	0.436	^*^0.0034
**ABCC1 Expression:**			**Vinblastine**
DNA Gene	*R*-value	−0.040	^*^0.429
Copy Number	*P*-value	0.383	^*^0.001
ABCC1 Expression	*R*-value	0.114	^*^0.398
(Microarray)	*P*-value	0.198	^*^0.003
ABCC1 Expression	*R*-value	0.134	0.299
(RT-PCR)	*P*-value	0.181	^*^0.036
**ABCG2 Expression:**			**Pancratistatin**
ABCG2 Expression	*R*-value	0.107	^*^0.329
(Microarray)	*P*-value	0.211	^*^0.006
ABCG2 Expression	*R*-value	0.048	^*^0.346
(Western Blot)	*P*-value	0.358	^*^0.004
**EGFR Expression:**			**Erlotinib**
EGFR Gene	*R*-value	0.049	−0.245
Copy Number	*P*-value	0.354	^*^0.029
EGFR Expression	*R*-value	0.144	^*^-0.458
(Microarray)	*P*-value	0.135	^*^1.15 × 10^−4^
EGFR Expression	*R*-value	−0.175	^*^0.409
(RNAse Protection)	*P*-value	0.094	^*^7.08 × 10^−4^
EGFR Expression	*R*-value	0.016	^*^-0.376
(Protein Array)	*P*-value	0.452	^*^0.001
**TP53 Mutation:**			**5-Fluorouracil**
TP53 Mutation	*R*-value	−0.198	^*^-0.502
(cDNA Sequencing)	*P*-value	0.068	^*^3.50 × 10^−5^
TP53 Function	*R*-value	−0.145	^*^-0.436
(Yeast Functional Assay)	*P*-value	0.148	^*^5.49 × 10^−4^
**pan-RAS Mutation:**			**Melphalan**
H-,K-,N-RAS Mutation	*R*-value	0.253	^*^0.367
	*P*-value	0.026	^*^0.002

* *R > 0.3 (or R < −0.3) and P < 0.05*.

### Drug class profiling

**Figure 2:**
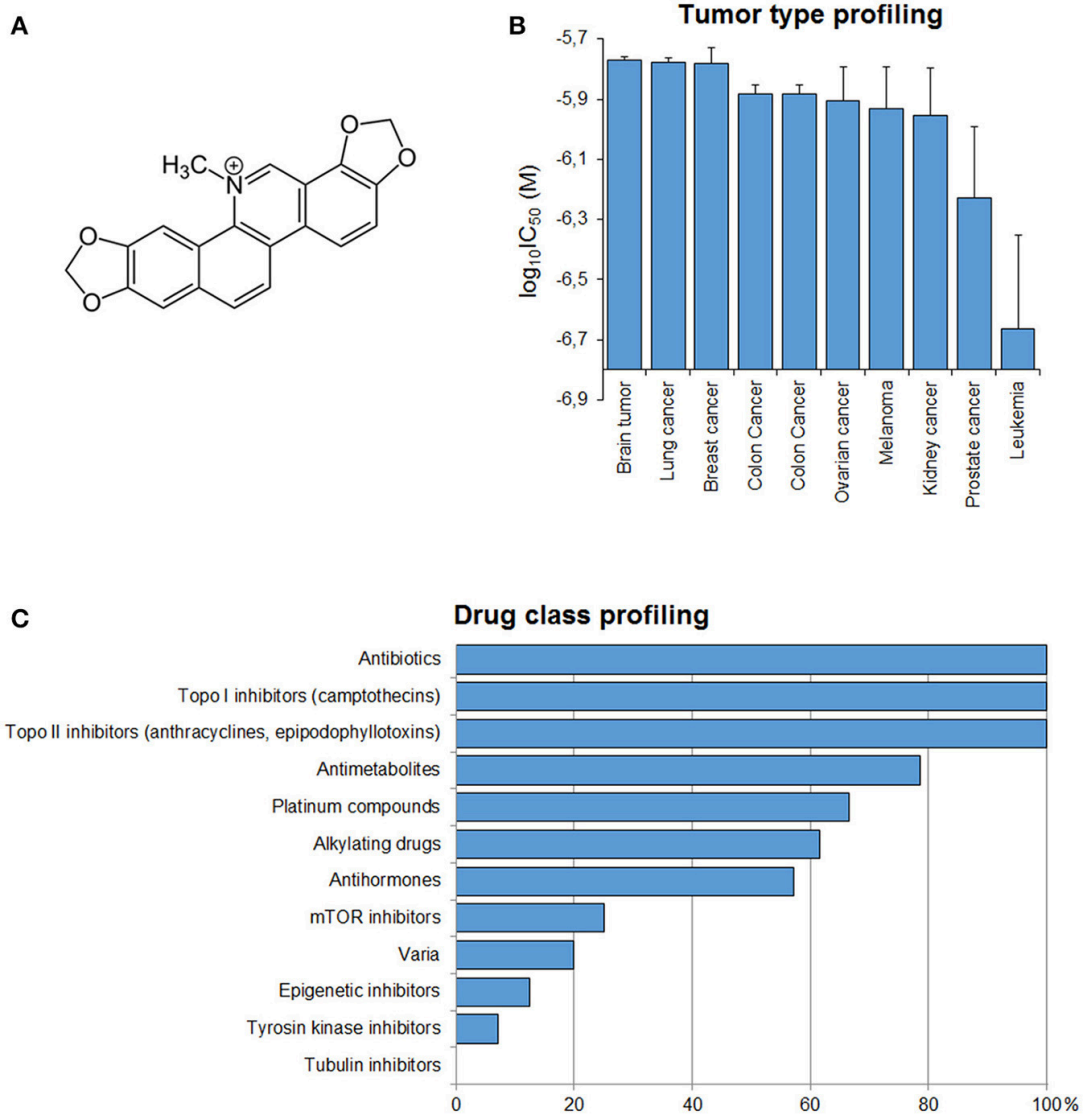
**(A)** Chemical structure of sanguinarine. **(B)** Mean log_10_IC_50_ values for sanguinarine of the NCI cell lines, mean values and S.E.M. of log10IC50 were grouped according to the tumor type of the cell lines. **(C)** Percentage of classes of established anticancer drugs, whose log_10_IC_50_ values correlate with those for sanguinarine.

To have a clue about the possible mechanism of action of sanguinarine, we correlated the log_10_IC_50_ values of the NCI cell lines to sanguinarine with those of standard drugs Figure 2C. The cellular response of antibiotics and DNA topoisomerase I or II inhibitors drugs significantly correlated with those of sanguinarine (100%). Antimetabolites and platinum compounds were correlated with sanguinarine (79 and 65%).

### Tumor-type dependent response toward sanguinarine

If the average of log_10_IC_50_ values of al cell lines according to their corresponding tumor type was plotted, it became apparent that leukemia cell lines were most sensitive toward snaguinarine, whereas brain cell lines were the most resistant (Figure 2B).

### Inhibition of P-glycoprotein function by sanguinarine

**Figure 3:**
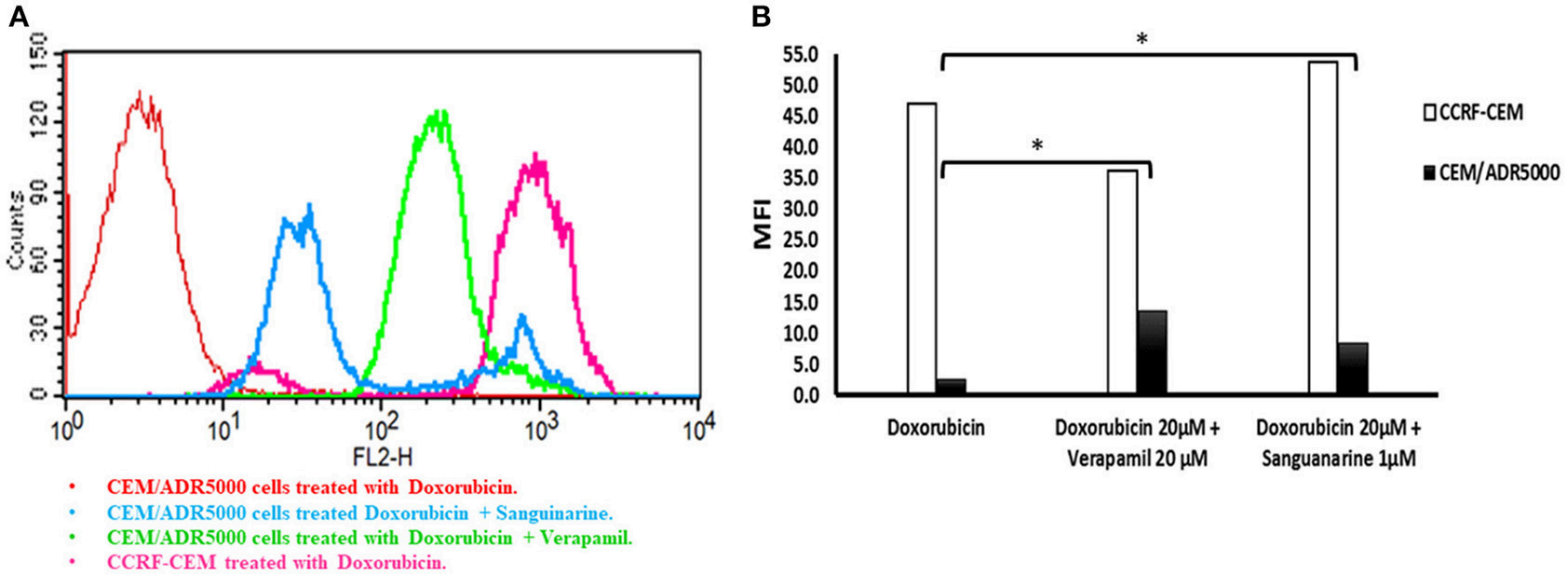
Flow cytometry analysis of the effect of sanguinarine on doxorubicin uptake by cell line that overexpress P-gp in comparison of known transporter's inhibitor, verapamil. **(A)** Histograms represent doxorubicin fluorescence intensity, CEM/ADR5000 cells treated with doxorubicin appear in red, CCRF-CEM cells treated with doxorubicin appears in pink, CEM/ADR5000 cells treated with doxorubicin in combination with sanguinarine and Verapamil appear in blue and light green respectively. **(B)** Bar diagram represent doxorubicin mean fluorescence intensity on cell line that overexpress P-gp, white bars represented sensitive cells, whereas resistant cells were represented by black bars. Mean fluorescence intensity (MFI) of doxorubicin has been measured by blue laser at excitation wave length 488 and emitted light was collected at 530/30 band pass. MFI values have been plotted on the y-axis against tested compounds on the x-axis. *P-value < 0.05.

The overexpression of P-glycoprotein in cancer cells mediates extrusion of chemotherapeutic drugs outside the cells leading to sub-lethal intracellular drug concentrations (Efferth, 2001). To maintain a high concentration of the chemotherapeutic drugs inside the cancer cells, P-gp has to be inhibited to enhance drug uptake. Therefore, we analyzed the uptake of doxorubicin by P-glycoprotein-overexpressing cells in the presence or absence of sanguinarine by flow cytometry. A representative histogram is shown in Figure 3A. High doxorubicin uptake was obtained in CCRF-CEM cells, whereas considerably less drug was taken up in CEM/ADR5000 cells due to P-glycoprotein-mediated efflux. The addition of sanguinarine (1 μM) or the well-known P-glycoprotein inhibitor verapamil (20 μM) increased doxorubicin uptake in CEM/ADR5000 cells. The bar diagram in Figure 3B shows that sanguinarine and verapamil significantly increased doxorubicin uptake in CEM/ADR5000 cells. The increase of doxorubicin uptake by P-glycoprotein-overexpressing cells after addition of sanguinarine or verapamil indicates that the transporter function in CEM/ADR5000 cells was specifically inhibited by both compounds.

### Microarray-based expression profiling to predict sensitivity and resistance to sanguinarine

To identify possible genes that are associated with sensitivity or resistance of cancer cells toward sanguinarine, we dug the transcriptome-wide mRNA expressions of the NCI cells and correlated them to the log_10_IC_50_ values for sanguinarine. We performed a transcriptome-wide COMPARE analysis to generate a ranking list of genes, whose mRNA expression directly or inversely correlated with the log_10_IC_50_ values for sanguinarine. Only cut-off values of correlation coefficients of *R* > 0.5 (direct correlations) or *R* < −0.5 (inverse correlations) were taken into account. Fourty genes were identified, half of them were directly and the other half were inversely correlated to the log_10_IC_50_ values for sanguinarine (Table 3). The proteins encoded by these genes have diverse biological functions (Table 3).

**Table 3:** Correlation of constitutive mRNA expression of genes identified by COMPARE analysis with log_10_IC_50_ values for sanguinarine of the NCI tumor cell lines.

**COMPARE coefficient**	**GenBank accession**	**Experimental ID**	**Gene symbol**	**Name**	**Function**
0.775	D86964	GC35690	DOCK2	Dedicator of cytokinesis 2	Functioning as a guanine nucleotide exchange factor (GEF), which exchanges bound GDP for free GTP
0.769	D89077	GC33424	SLA	Src-like-adaptor RNA	Negatively regulates T-cell receptor (TCR) signaling
0.765	AA133246	GC36535	LAIR2	Leukocyte-associated immunoglobulin-like receptor 2	It was identified by its similarity to leukocyte-associated immunoglobulin-like receptor 1
0.76	D14889	GC33186	RAB33A	RAB33A, member RAS oncogene family	It is GTP-binding protein and may be involved in vesicle transport
0.759	AB007918	GC35631	KIF21B	Kinesin family member 21B	Is involved in regulation of microtubule dynamics, synapse function and neuronal morphology, including dendritic tree branching, and spine formation
0.748	D30758	GC27814	ACAP1	ArfGAP with coiled-coil, ankyrin repeat and PH domains 1	GTPase-activating protein (GAP) for ADP ribosylation factor 6 (ARF6) required for clathrin-dependent export of proteins from recycling endosomes to trans-Golgi network and cell surface
0.718	M37033	GC28789	CD53	CD53 molecule	Required for efficient formation of myofibers in regenerating muscle at the level of cell fusion
0.715	AB018254	GC39476	KBTBD11	Kelch repeat and BTB (POZ) domain containing 11	Unknown
0.711	U51127	GC32375	IRF5	Interferon regulatory factor 5	Transcription factor involved in the induction of interferons IFNA and INFB and inflammatory cytokines upon virus infection
0.71	AF013249	GC27873	LAIR1	Leukocyte-associated immunoglobulin-like receptor 1	It also reduces the increase of intracellular calcium evoked by B-cell receptor ligation. May also play its inhibitory role independently of SH2-containing phosphatases
0.709	X93595	GC27133	KIR3DL1	Killer cell immunoglobulin-like receptor, three domains, long cytoplasmic tail, 1	Receptor on natural killer (NK) cells for HLA Bw4 allele. Inhibits the activity of NK cells thus preventing cell lysis
0.699	AJ243937	GC29466	GPSM3	G-protein signaling modulator 3	GTPase regulator activity and GDP-dissociation inhibitor activity
0.699	AJ008112	GC27500	FMNL1	Formin-like 1	May play a role in the control of cell motility and survival of macrophages
0.696	U63127	GC31882	PSD4	Pleckstrin and Sec7 domain containing 4	Through ARL14 activation, controls the movement of MHC class II-containing vesicles along the actin cytoskeleton in dendritic cells
0.695	AF031824	GC37973	CST7	Cystatin F (leukocystatin)	Inhibits papain and cathepsin L but with affinities lower than other cystatins
0.694	D87457	GC38648	ELMO1	Engulfment and cell motility 1	Required in complex with DOCK1 to activate Rac Rho small GTPases. May enhance the guanine nucleotide exchange factor (GEF) activity of DOCK1
0.692	L41268	GC27284	KIR2DL3	Killer cell immunoglobulin-like receptor, two domains, long cytoplasmic tail, 3	Receptor on natural killer (NK) cells for HLA-C alleles (HLA-Cw1, HLA-Cw3, and HLA-Cw7). Inhibits the activity of NK cells thus preventing cell lysis
0.69	Y00638	GC30951	PTPRC	Protein tyrosine phosphatase, receptor type, C	Upon T-cell activation, recruits and dephosphorylates SKAP1 and FYN
0.685	J04132	GC27477	CD247	CD247 molecule	Probable role in assembly and expression of the TCR complex as well as signal transduction upon antigen triggering
0.685	U73394	GC28642	KIR2DL4	Killer cell immunoglobulin-like receptor, two domains, long cytoplasmic tail, 4	Inhibits the activity of NK cells thus preventing cell lysis
−0.506	N36926	GC31915	GNA11	Guanine nucleotide binding protein (G protein), alpha 11 (Gq class)	Acts as an activator of phospholipase C
−0.487	M22299	GC37799	PLS3	Plastin 3	May play a role in the regulation of bone development
−0.472	AB011159	GC36349	NCKAP1	NCK-associated protein 1	Part of the WAVE complex that regulates lamellipodia formation
−0.45	D84454	GC37231	SLC35A2	Solute carrier family 35 (UDP-galactose transporter), member A2	Transports nucleotide sugars from the cytosol into Golgi vesicles where glycosyltransferases function
−0.449	M35011	GC34135	ITGB5	Integrin, beta 5	Acts as a receptor for adenovirus type C
−0.435	D10522	GC35417	MARCKS	Myristoylated alanine-rich protein kinase C substrate	This protein binds calmodulin, actin, and synapsin. MARCKS is a filamentous (F) actin cross-linking protein
−0.431	AF102265	GC29686	PGM3	Phosphoglucomutase 3	Catalyzes the conversion of GlcNAc-6-P into GlcNAc-1-P
−0.431	AL050268	GC37394	RAB1A	RAB1A, member RAS oncogene family	RAB1A regulates vesicular protein transport from the endoplasmic reticulum (ER) to the Golgi compartment and on to the cell surface, and plays a role in IL-8 and growth hormone secretion
−0.428	L10284	GC30552	CANX	Calnexin	Calcium-binding protein that interacts with newly synthesized glycoproteins in the endoplasmic reticulum
−0.422	U57627	GC37169	OCRL	Oculocerebrorenal syndrome of Lowe	Membrane trafficking by regulating the specific pool of phosphatidylinositol 4,5-bisphosphate that is associated with lysosomes
−0.419	S71018	GC27825	PPIC	Peptidylprolyl isomerase C (cyclophilin C)	It catalyzes the cis-trans isomerization of proline imidic peptide bonds in oligopeptides
−0.414	AB007860	GC29831	ASAP2	ArfGAP with SH3 domain, ankyrin repeat and PH domain 2	Modulates PXN recruitment to focal contacts and cell migration
−0.412	AF089816	GC38772	GIPC1	GIPC PDZ domain containing family, member 1	May be involved in G protein-linked signaling
−0.412	L13857	GC36251	SOS1	Son of sevenless homolog 1 (Drosophila)	Promoting Ras activation, regulates phosphorylation of MAP kinase MAPK3 in response to EGF
−0.409	AF019612	GC34390	MBTPS2	Membrane-bound transcription factor peptidase, site 2	Intramembrane proteolysis of sterol-regulatory element-binding proteins
−0.409	AF035287	GC38763	NPTN	Neuroplastin	May play a role in synaptic plasticity
−0.409	L10678	GC29255	PFN2	Profilin 2	Binds to actin and affects the structure of the cytoskeleton
−0.404	AF017445	GC28548	TNNI3K	TNNI3 interacting kinase	May play a role in cardiac physiology
−0.403	AL049943	GC36426	FAM98A	Family with sequence similarity 98, member A	Unknown
−0.402	M36341	GC26981	ARF4	ADP-ribosylation factor 4	GTP-binding protein that functions as an allosteric activator of the cholera toxin catalytic subunit, an ADP-ribosyltransferase

**Figure 4:**
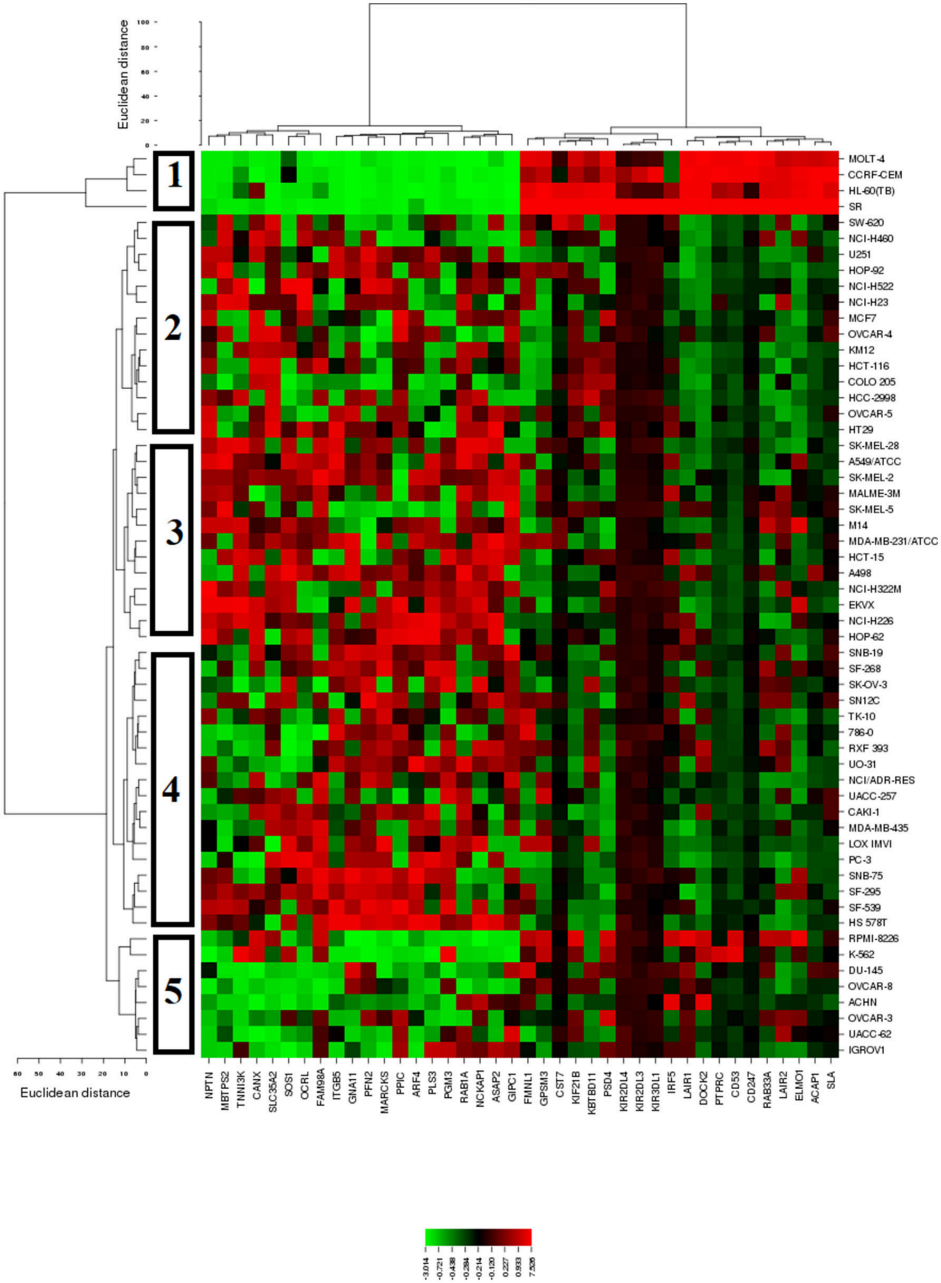
Dendrograms and heatmap of sanguinarine obtained by hierarchical cluster analyses of NCI cells line panel and genes whose mRNA expression directly or inversely correlated with the log_10_IC_50_ values for sanguinarine. The dendrogram on the left shows the clustering of cell lines and the dendrogram on the top shows the clustering of genes.

The mRNA expression values of all NCI cell lines for the genes listed in Table 3 were subsequently subjected to hierarchical cluster analysis, in order to find out, whether clusters of cell lines could be identified with similar behavior after exposure to sanguinarine. The dendrogram of the cluster analysis showed five main branches in the cluster tree that depicted in the heatmap (Figure 4). As a next step, the log_10_IC_50_ values for sanguinarine, which were not included in the cluster analysis, were assigned to the corresponding position of the cell lines in the cluster tree. The distribution among the five clusters was significantly different from each other (*P* = 0.03). Clusters 4 and 5 contained in its majority of cell lines resistant to sanguinarine, whereas clusters 1, 2, and 3 contained in its majority sensitive ones. The median value of the log_10_IC_50_ values was used as a cutoff value to define cell lines as being sensitive or resistant to sanguinarine.

### Transcription factor gene promoter binding motif analysis

**Figure 5:**
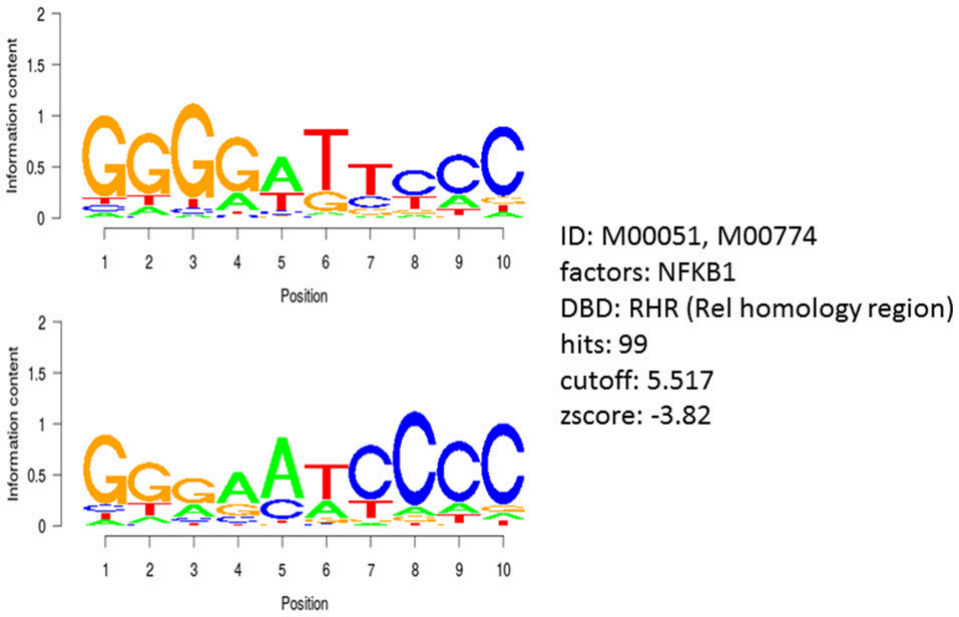
Motif analysis of 25 kb upstream regions of 40 genes identified by COMPARE analysis revealing the significant presence of NF-κB binding motif.

We subjected the 40 genes identified by COMPARE analysis to binding motif analysis to find possibly common regulatory transcription factors. The theoretical concept behind was that, a wide array of functionally diverse genes might be commonly up-or down-regulated by one or a few transcription factors that are involved in resistance to cytotoxic compounds. Interestingly, the NF-κB DNA binding motif (Rel) was widely distributed in the untranslated promoter regions 25 kb upstream of all identified genes, with 99 hits and a Z-score of −3.8 (Figure 5). This analysis pointed out that NF-κB might play a considerable role in the regulation of genes associated with cellular response to sanguinarine.

### Molecular docking

**Figure 6:**
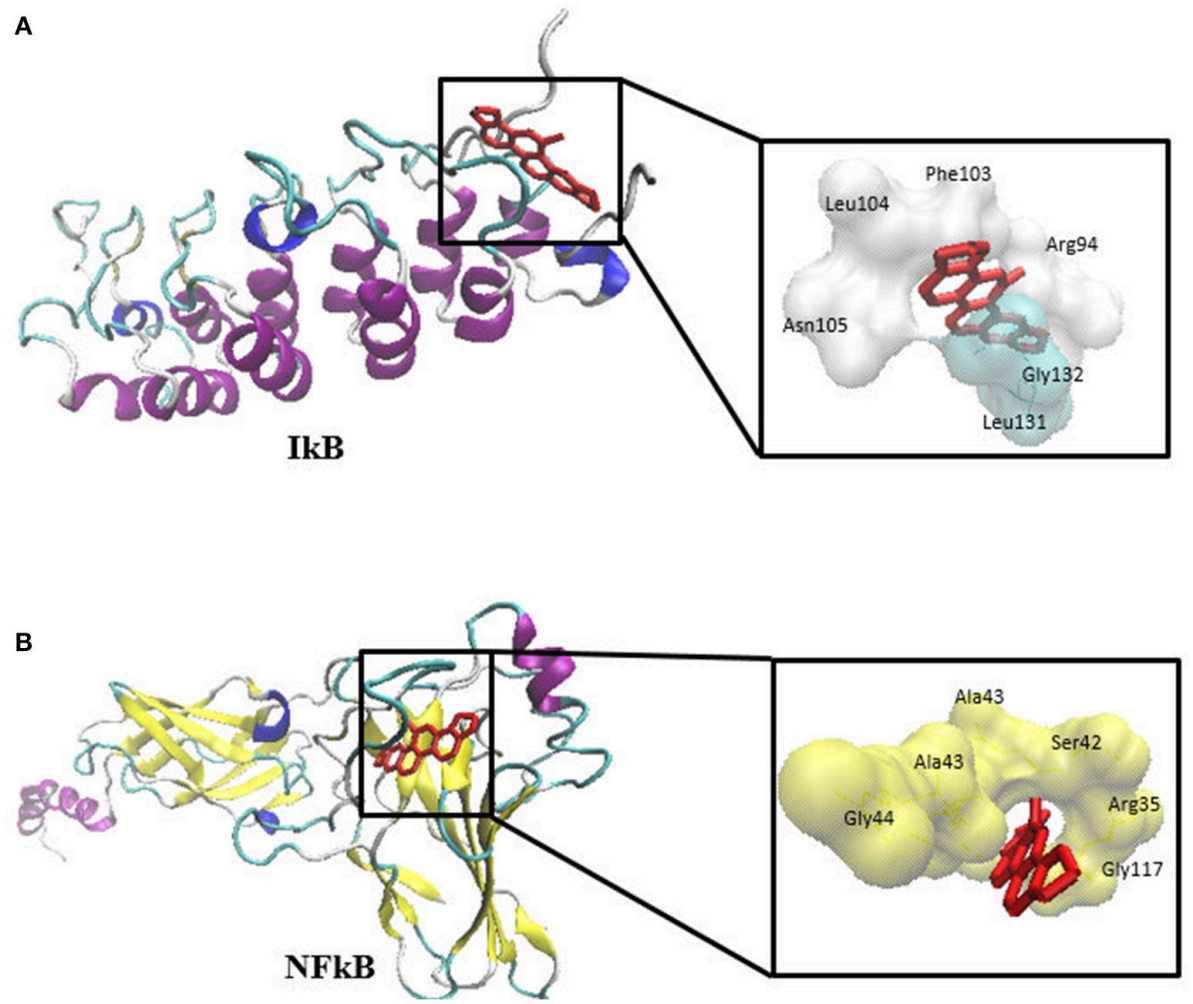
Molecular docking of sanguinarine to **(A)** IκB and **(B)** NF-κB proteins.

The previous results allow speculating that sanguinarine might prevent the degradation of IκBα leading to maintenance of the IκB-NF-κB complex in its idle form (Chaturvedi et al., 1997). In order to substantiate this idea, we performed molecular docking analyses studying the binding affinity of sanguinarine to both proteins. As shown in Table 4, sanguinarine preferably bound to IκBα with a binding energy of −8.2 kcal/mol, whereas sanguinarine exerted lower affinities to NF-κB with a binding energy of −7.2 kcal/mol. These results indicate that sanguinarine has a higher affinity to IκB than to NF-κB. The corresponding docking positions of sanguinarine into binding pockets of IκB and NF-κB are depicted in Figure 6.

**Table 4:** Molecular docking of sanguinarine to IκB and NF-κB proteins.

**Proteins**	**Lowest binding energy kcal/mol**	**Pki (μM)**	**H-bond**	**Hydrophobic interaction**
IκB	−8.17 ± <0.001	1.03 ± <0.001	Ile94	Leu104, Phe103, Asn105, Gly132, Leu131, Val93, Arg95
NF-κB	−7.27 ± 0.02	4.67 ± 0.19	Ile118	Gly117, Arg35, Ser42, Gly44, Ala43, Arg41

*Shown are lowest binding energy, predicted inhibition constant (Pki), amino acids (AA) involved in hydrogen bonding, and hydrophobic interaction. Each docking experiment has been repeated three times*.

### NF-κB reporter cell line

**Figure 7:**
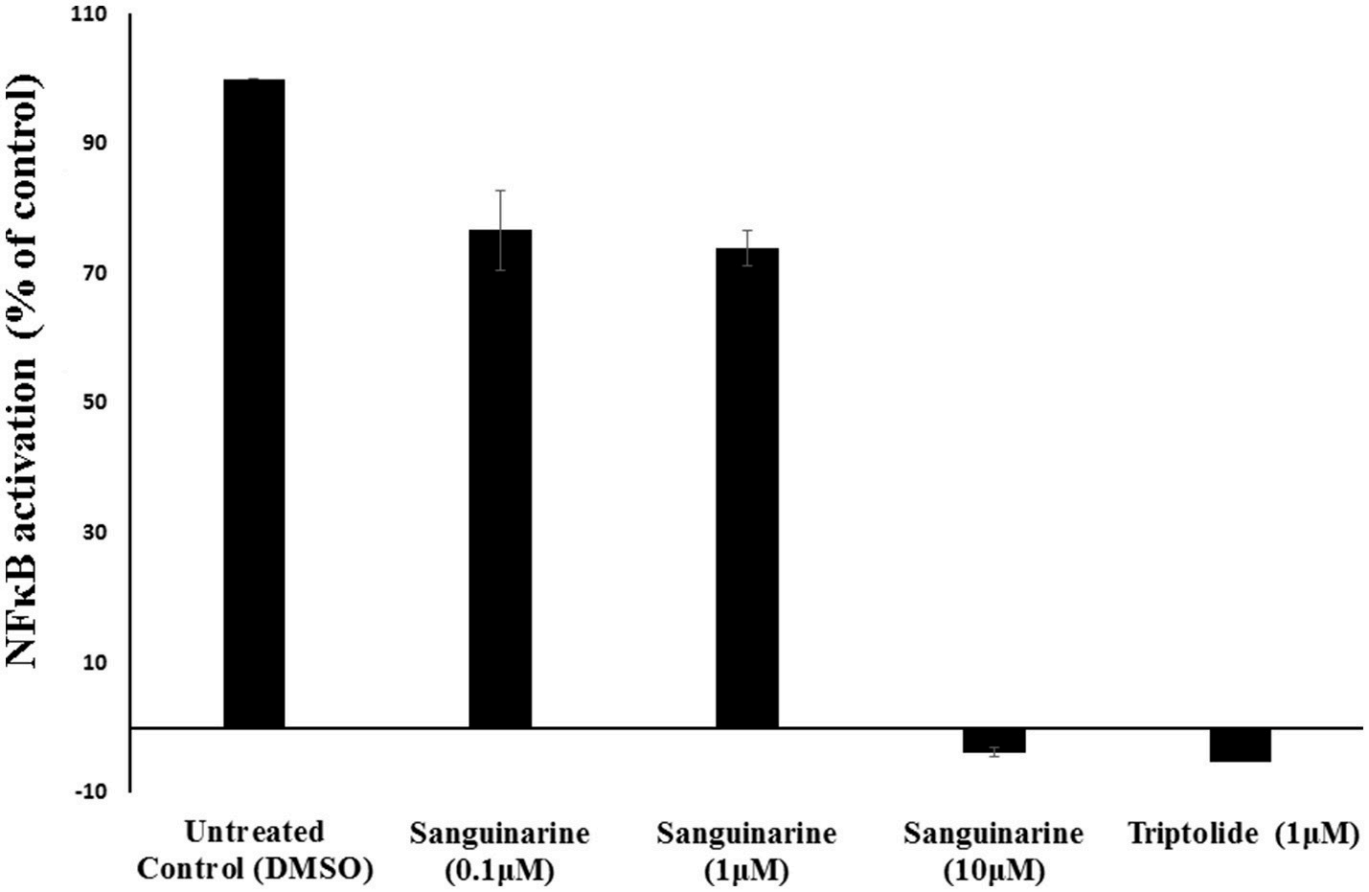
Effect of sanguinarine on NF-κB activity in comparison to the known NF-κB inhibitor, triptolide, as positive control.

Molecular docking studies and motif analysis have predicted the ability of sanguinarine to interact with NFκB and it's regulator IκB. However, since these are speculative findings we could not reach a clear statement whether sanguinarine inhibits or activates the NFκB. Therefore, we performed NFκB reporter assay using SEAP-driven cell line to address this question. HEK-Blue-Null1 cells were treated with varying concentrations of sanguinarine (0.1, 1, and 10 μM). A known inhibitor of NFκB activation, triptolide (1 μM), was used as a positive control. Interestingly, sanguinarine reduced the SEAP levels in a dose dependent manner, indicating that NFκB's activity is blocked Figure 7. This result clearly showed that sanguinarine is an inhibitor of NFκB activity.

### Immunoblotting analysis

**Figure 8:**
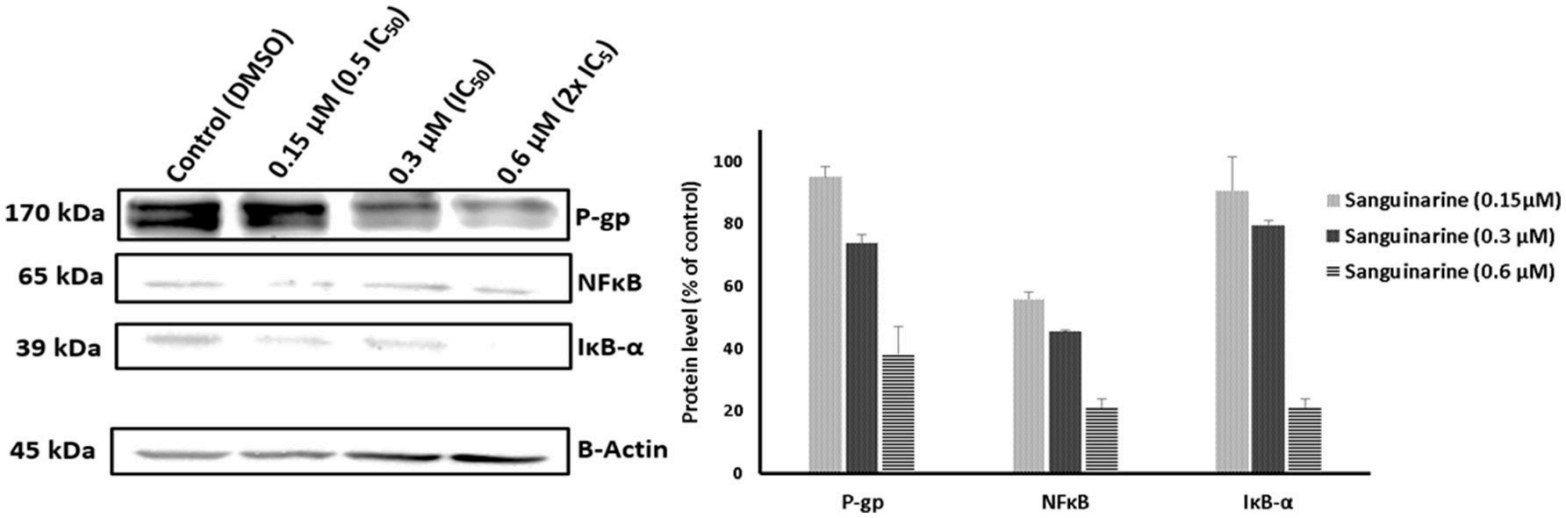
Western blot analysis of the effect of Sanguinarine on CEM/ADR5000 leukemia cells. Evaluation of the P-gp, NFκB, and IκBα expressions. β-actin was used as loading control. Bands were normalized to β-actin in order to obtain numerical values (Mean ± *SD*).

We have a clue that the inhibition of NκFB activity may explain the phenomenon of the collateral sensitivity of resistant P-gp overexpressing cells toward sanguinarine. To assess this hypothesis, the P-gp, NFκB, and IκBα expressions were evaluated by immunoblotting analysis. Three concentrations of sanguinarine were selected (0.5 IC_50_, IC_50_ and 2x IC_50_). Interestingly, the expressions of all three proteins were decreased in a dose dependent manner (Figure 8).

## Discussion

Faced with the general requirement to develop novel cancer drugs with activity against otherwise drug-resistant tumors, we focused on sanguinarine, a natural product from *S. canadensis*. In the present study, sanguinarine revealed considerable cytotoxicity against several drug-resistant tumor cell lines. The cytotoxic concentrations needed to kill 50% of the cell lines tested were all in the micromolar range. While sanguinarine's cytotoxicity against cancer cells has been previously reported (Ding et al., 2002; Debiton et al., 2003; Choi et al., 2008), it was not known whether or not sanguinarine's activity may be hampered by mechanisms that also confer resistance to clinically established anticancer drugs. Therefore, the main aim of our study was to investigate the capability of sanguinarine to kill otherwise drug-resistant tumor cells. For this reason, we used cell lines overexpressing drug-resistance-conferring ABC-transporters (P-gp/*ABCB1, BCRP*, or *ABCB5*), or harboring mutations in the *EGFR, RAS*, or *TP53* genes.

ABC transporters are responsible for chemotherapy failure by expelling drugs outside of tumor cells and, thereby, minimizing doses required for cells killing. From the 49 members of the ABC transporter family, P-gp and BCRP were extensively studied for their contribution to MDR (Ueda et al., 1986; Doyle et al., 1998). A few years ago, a new member of the B-subfamily conferring MDR had been cloned, ABCB5 (Frank et al., 2003). This transporter is closely related to P-gp and exhibits resistance to doxorubicin, paclitaxel, and docetaxel in cancer cells (Frank et al., 2005; Kawanobe et al., 2012). All our cell models that overexpress the ABC transporters mentioned above did not confer resistance to sanguinarine indicating that this compound is not a substrate for these transporters. Interestingly, sanguinarine showed hypersensitivity (collateral sensitivity) to P-gp-overexpressing cells, if compared to their parental drug-sensitive counterparts. From a therapeutic point of view, collateral sensitivity is a favorable phenomenon, since the corresponding cytotoxic agent preferentially kills drug-resistant cells (Hall et al., 2009). This finding led us to the assumption that sanguinarine may interfere with P-glycoprotein's function. Therefore, we performed doxorubicin uptake assays. Indeed, sanguinarine comparably increased doxorubicin uptake, as the known P-gp inhibitor, verapamil, did. This is a remarkable result, since verapamil is not only an inhibitor of drug efflux in P-glycoprotein, it also exerts collateral sensitivity if applied alone (Pluchino et al., 2012). In this respect, sanguinarine behaves comparably as verapamil.

EGFR is a member of the HER family. Upon binding of its ligands, EGF or TGF-α, it forms homo- or hetero-dimers with other HER family members to activate specific downstream signaling cascades after tyrosine phosphorylation. This activation of signaling routes subsequently regulates multiple cellular processes, such as proliferation, survival, and apoptosis. EGFR mutations are known to affect the poor prognosis of patients and mediate drug resistance of tumors. The in-frame deletion of the extracellular EGFR domain results in ligand-independent receptor activations and represents a common mutant type in brain tumors, named ΔEFGR (Shinojima et al., 2003). Sanguinarine inhibited both cell models harboring wtEGFR (sensitive) or ΔEFGR (resistant) at the same concentrations, indicating that ΔEFGR does not confer resistance toward sanguinarine.

P53 is a transcription factor that regulates the expression of p21^WAF1/CIP1/SID1^, MDM2, Bax, and Gadd45. These proteins play a critical role in cell cycle arrest, DNA repair and induction of apoptosis. P53 mutations occur in almost half cancer types ever known (Hollstein et al., 1991), and loss of p53 function is linked to drug resistance in several tumor entities, including carcinoma of the breast, ovary, and colorectum as well as melanoma, acute lymphoblastic leukemia, neuroblastoma and osteosarcoma (Li et al., 1998; Asada et al., 1999; Lam et al., 1999; Righetti et al., 1999; Berns et al., 2000). Cells lacking alleles of p53 (HCT116 p53^−/−^) conferred resistance to 5-FU, irinotecan and oxaliplatin (Boyer et al., 2004). Unexpectedly, our results showed that sanguinarine preferentially inhibited the resistant p53 knockout colorectal cancer cells comparably to the cells bearing wt p53. This result indicates that sanguinarine may have another molecular target, which is functionally regulated in the presence or absence of p53. Worth to mention, Webster et al., proved that both p53 and NF-κB inhibited each other's ability to stimulate gene expression by competing for the limited pool of transcriptional co-activator p300 and CREB-binding protein (Webster and Perkins, 1999). This means NF-κB inhibits p53-dependent transactivation, whereas p53 expression down-regulates NF-κB expression leading to diminished drug resistance and prevention of apoptosis and tumorigenesis. Moreover, they concluded that reactivation of wild-type p53 function by coordinated suppression of NF-κB might favor a better clinical outcome. Recently, several studies have affirmed that mutant p53 augmented and prolonged NFκB activation *in vitro* and *in vivo* (Scian et al., 2005; Weisz et al., 2007; Schneider et al., 2010; Cooks et al., 2013). Apparently, all these studies gave us a clue that NF-κB might be the target of sanguinarine. Therefore, transcription factor gene promoter binding motif analysis for genes appeared from wide transcriptome microarray data have been carried out. Intriguingly, the NF-κB binding motif was detected in the upstream promoter regions of all genes, confirming that NF-κB may be a master transcription regulator for most—if not all—molecular determinants of sanguinarine's activity in cancer cells.

NF-κB is an ubiquitous transcription factor that regulates the expression of genes involved in inflammation, the immune response, cell proliferation, and apoptosis (Li and Verma, 2002). It is inactive in the cytoplasm bound to its inhibitory protein, IκB. Upon activation by cytokines, IκB subsequently undergoes phosphorylation, ubiquitination, and degradation by the proteasome allowing nuclear translocation of NF-κB (Baldwin, 1996). Sanguinarine has been found to inhibit phosphorylation and degradation of IκB keeping NF-κB in its inactive form (Chaturvedi et al., 1997). This result concurs well with our molecular docking analyses, which showed that sanguinarine has high affinity to IκB compared to NF-κB. Moreover, a recent study reported that PI3K/Akt/NF-κB pathway is involved in the development of MDR in MCF-7/ADR cells through the upregulation of the MDR1 gene and P-gp expression. Accordingly, the MDR could be reversed in P-gp overexpressing cells through inhibition of the PI3K/Akt/NF-κB signaling pathway (Yang et al., 2017). Taking Yang et al.'s study together with our immunoblotting analysis results (Figure 8) into account, these findings gave us clear evidence that sanguinarine not only inhibits the activity of P-gp but also downregulates its expression via inhibition of NFκB.

The COMPARE analysis explores the relationship between the gene expression patterns and the drug responsiveness measured by the NCI developmental therapeutic program. The patterns of drug activity across the 60 NCI cell lines provide a clue on mechanisms of drug action, resistance and sensitivity determinants (Ross et al., 2000). The approach of defining genes whose mRNA expression correlated with sanguinarine's activity is based on Pearson correlation coefficient. These correlation coefficients (Table 3) were calculated for each combination of a gene and a drug by taking the (normalized) level of expression of the gene in each cell line, multiplying it by the corresponding (normalized) sensitivity of the cell to the drug, summing the results over all of the cell lines and renormalizing (Scherf et al., 2000). By combining genome-wide expression profiling with sanguinarine's activity data, a possible genes-drug interaction was explored. This comprehensive approach allowed us to assess information of a large number of genes simultaneously and generate hypotheses about the possible mode of action rather than testing a particular biological hypothesis classically, in which cell characteristics were assessed one gene, gene product or molecular pathway at a time (Weinstein, 1998). The theme of the cluster analysis was to group cell lines depending on the levels of gene expressions which correlated to sanguinarine's activity. The log_10_IC_50_ values were excluded from the calculation. As shown in Figure 4, leukemia cells were clustered as the most sensitive ones. Interestingly, this predictive findings harmonized with our *in vitro* cytotoxic assays in which small IC_50_ values were obtained on leukemia cells after sanguinarine treatment when compared to other tested solid tumor cells (brain, breast, and colon).

The COMPARE analysis revealed that most of the identified genes are involved in activating the immune response and promoting inflammatory signaling (*Dock2, SLA, LAIR2, IRF5, KIR3DL1, FMNL1, PSD4, KIR2DL3, PTPRC, CD247, KIR2DL4, LAIR1, ITGB5, RAB1A*, and *ARF4*). Thus, it is not a coincidence that a prototypical proinflammatory protein such as NF-κB appeared as a presumable master regulator of transcription of genes recognized in microarray-based transcriptomic mRNA expression data. Recently, Wang et al., proved that sanguinarine as anti-inflammatory agent prevented cerebral stroke *in vivo* via downregulation of the inflammatory cytokines, TNF-α, IL-1β and IL-6 (Wang et al., 2017). Moreover, other genes from diverse functional groups appeared in our analysis, such as signal transducers promoting cancer growth (*CD53, RAB33A, GNA11, CANX, OCRL, GIPC1*, and *SOS1*), microtubule formation (*KIF21B*) and cell migration (*ASAP2*). These cellular processes may be of specific relevance for the responsiveness of tumor cells to sanguinarine.

In conclusion, sanguinarine exhibited remarkable cytotoxicity against different drug-resistant tumor cell lines, which is possibly dependent on multiple mechanisms. The increased doxorubicin uptake by resistant cells indicates that sanguinarine inhibited the P-glycoprotein transporter. Furthermore, sanguinarine inhibited NF-κB activation. In particular, sanguinarine may be a promising candidate either if used alone or in combination with other chemotherapeutic regimens to treat refractory tumors. Nevertheless, the safety profile of sanguinarine should be well investigated, and the chemical scaffold could be further derivatized to increase the therapeutic index. Therefore, further pharmacological studies are needed.

## Author contributions

MS: Carried out the *in vitro* experiments and wrote the manuscript; NM: Performed COMPARE and cluster analysis; YS: Provided ABCB5 transfected cell lines; HA-A: Designed the bioassays and edited the manuscript; TE: Supervised the work, provided the facilities for the study and edited the manuscript. All authors read the manuscript and approved the final version.

### Conflict of interest statement

HA-A was employed by company by Bayer/Steigerwald. The other authors declare that the research was conducted in the absence of any commercial or financial relationships that could be construed as a potential conflict of interest. The reviewer WZ and handling Editor declared their shared affiliation.
